# Rain and Rheumatism

**Published:** 1880-07

**Authors:** 


### RAIN AND RHEUMATISM.

The usual arrangements had been fully completed for our annual camping out expedition. Tents, cooking utensils, provisions, everything, packed and the dray on the barn floor loading, preparatory to carrying them to the depot. It was Monday morning. But an hour intervened to train time. I had awakened that very morning with an excruciating pain in the joint of my great-toe. So persistent was the pain that I was compelled to resort to crutches to enable me to superintend the loading of our fixtures. The campers were all on hand eager to go, but none of them more so than I. The swelling continued in the toe-joint however, and by the time the dray was ready to leave the yard, I was completely prostrated with the rapidly increasing pain. It soon became evident that I must yield to the solicitations of friends and postpone the day for our departure. This was exasperating and pained me almost as much as the confounded toe. Friends from New York, Brooklyn. Philadelphia and Washington were expected in camp before the week would end; indeed, two friends had already left New York for the camping grounds. There was no way of heading them off, hence my great perplexity. At last, a good friend came to my relief and volunteered to go to the camping grounds and bring them to my house. This settled, I went to bed where I was forced to remain for nearly two weeks, with the most painful of all diseases —inflammatory rheumatism.

At last, I was able to hobble about by aid of a cane, and at once gave orders for our departure; and here I am, on the Lycoming, the same delightful spot upon which we have camped regularly for the past nine years.

We landed at the station in a thunder storm, and put up our tents between showers. I took a position under a great beech, and, while the rain trickled down my spine, gave directions about the arrangement of the camp, for I was too lame to take any more active part.

The natives of this valley have learned to pray for our coming, for, with us invariably comes also the rain. Now, rain is indispensable to farmers, but to campers and rheumatics it is not regarded as a necessity nor even desirable. But, having passed an entire week here with an abundance of rain daily, I have come to believe that rain and rheumatism are not incompatible. I have slept soundly between damp sheets and have laid my head upon a wet pillow while the mist from the falling rain upon the tent has sprayed my face, yet, have awakened every morning feeling better, until at this writing, I am able to take my rod and supply my quota of trout for the breakfast table.

I have sat, with my inflamed foot upon a camp stool, under a sheltering canopy, where the dripping trees sent great drops of rain through the canvas to spatter my paper, and have watched the birds hump their backs and squirrels whisk their tails to prevent the rain from penetrating to their skins. I don't believe a bird enjoys the rain any more than do the campers. Certainly, that cat-bird, perched on a limb over-head, does not. An hour ago, he was lively as a cricket and seemingly very happy, warbling and whistling as he hopped cheerily from limb to limb, watching with much interest the mysterious movements of the invaders of his domain beneath. But, the moment he heard the thunder pealing over the mountain beyond our view, he cocked one eye heavenward, winked with the other one, dropped his tail and became motionless and silent as an oyster. Not another note has he uttered ; and now, there he sits as morose as a dyspeptic, his back arched, his feathers drawn close, and occasionally shakes his head to free his eyes from the blinding rain. Just once has he deigned to look down at me to see how I am getting along, and I am sure is prepared to say, if he only could, "confound the rain."

Poor fellow! Wonder if he has ever had rheumatism in his great toe, and what his opinion is of the advisability of such a sufferer sitting out in a rain storm, just for fun.

Yes, here I sit, watching nature in her different moods and waiting for the appearance of fairer skies. I note the constantly varying colors of the foliage upon the steep mountain, at the foot of which our tents are pitched. Before us, a clear mountain stream, rock bound and musical as tinkling bells. Above the rocks, rhododendrons, profuse in their blossoms that peep out from the overhanging foliage of the great trees beyond, as though Nature had constructed a delightful boquet upon a grand scale. As I gaze upon and admire the scene, constantly changing my position to keep the rheumatic portion of my person out of the wet, the thunder rolls over head and the great black clouds shut out the light, changing all the hues in my great boquet. Now, flashes of lightning illuminate it with a new and weird brilliancy, revealing the very moss covered rocks at the foot of the trees. Soon, the storm passes over and beyond the great mountain, the sun peeps out from behind a lagging cloud, again lighting up and warming our camp, putting everyone in good humor. The birds are the first to give utterance to their appreciation of the change and make the mountain musical with their wild and ecstatic warblings. The catbird is one of the first to express his joy and is even now chirruping and whistling as though in an endeavor to outdo all his feathered friends.

An hour has passed and so has all traces of the storm. The wind has shaken the rain drops from the trees and the warm sun has dried the ground. The birds are singing merrily, the bees humming among the flowers, and the butterflies flying in zig-zag courses about the camp, causing everything to appear cheerful, and dispelling all thoughts of storms. Placing an arm chair—constructed of a barrel and piece of jute—in one of the boats, I paddle down to the great rock in the pond, anchor the boat under the shade of a tree, bait a hook and bob for suckers. A sucker is slow to make up his mind as to whether he had better partake of any tempting morsel offered him, so I have provided myself with a novel, in the perusal of which I may kill time as well as suckers. I find this very pleasant and engaging amusement for a contemplative rheumatic mind, for, one's attention becomes about evenly divided between the bobbing cork and the startling situations in which the characters in the novel are found. Just now, as the heroine was preparing to escape from a prison window by means of a rope provided by the ardent lover without, and as my mind outran the story and foresaw what a splendid time the two lovers would have hugging each other as they slid down that rope; down went my cork, followed by the novel, also the heroine, to give me an opportunity to jerk on that rod, which I did right vigorously and was rewarded with a monstrous, solemn looking sucker who continually extended his proboscis in derision or dismay at the situation. While I was thus engaged with the sucker, the lovers slid down the rope unobserved and got nicely away to a magnolia grove. It was moonlight to them, but broad day-light to me and the sucker. The young man had his arm about the damsel and was pouring all sorts of sweet sentences into her willing ear. They again had plighted their love and were making preliminary preparations to seal with a kiss. My mind was fully wrought up to the occasion. I imagined even that it was dark, (notwithstanding the sun was scorching my nose,) smelled the magnolias, saw the pretty girl and her pouting lips, indeed the smack had almost saluted my ears, when down went that confounded cork again. I could'nt help it, I just *had* to jerk on the pole and delivered in the boat a squirming, slippery eel! Was there ever before such a discordant mingling of fiction and reality! Sentiment and eels! I contemplated that slimy, squirming creature in the bottom of the boat, for ten minutes, trying to reconcile him with that love story, but he would'nt mix. I never saw a young woman that would fraternise with an eel, and fearing that the young man might lose his kiss, I threw the luscious villian overboard and resumed my story, only to find that he did not kiss her after all—something had alarmed them and they had fled while I was fussing with the blamed eel. Having lost the felicity of witnessing the lover's salute and being cheated out of my prospective breakfast, I permitted the lovers to continue their flight while I paddled back to camp, queer phantoms of suckers, eels, lovers, pouting lips, painful toes, and fair maidens leading the way.

Between showers, I sit rubbing a soothing liniment upon that aching toe, dry my back in the sunshine, and indite this epistle to the readers of the BISTOURY, assuring them that a chap with rheumatism may camp out if he will, and prepare myself for another rain.

